# Development of ionic polymers: novel [SPVP]TfO-graphene nanocomposites for sustainable water treatment and enhancing dye removal efficiency

**DOI:** 10.1039/d6ra01404f

**Published:** 2026-04-08

**Authors:** Mahmoud A. Hussein, Layla R. Alfahmy, Dina A. Bakhotmah, Mohamed A. Abdel-Fadeel, Tamer S. Saleh, Salsabeel Al-Sodies

**Affiliations:** a Chemistry Department, Faculty of Science, King Abdulaziz University P.O. Box 80203 Jeddah 21589 Saudi Arabia mahussein74@yahoo.com maabdo@kau.edu.sa; b Chemistry Department, Faculty of Science, Assiut University Assiut 71516 Egypt; c Department of Chemistry, College of Science, University of Jeddah Jeddah 80327 Saudi Arabia; d Department of Chemistry, Faculty of Science, Taibah University Al-Madinah Al-Munawarah 30002 Saudi Arabia

## Abstract

The synthesis and application of a novel nanocomposite material for dye removal from aqueous solutions is described in this work. The nanocomposite consisted of sulfonated poly(vinylpyrrolidonium) triflate [SPVP]TfO reinforced with graphene nanosheets (G). Various concentrations of graphene (0.2–10 wt%) were incorporated into the [SPVP]TfO matrix *via in situ* polymerization. The resulting nanocomposites were extensively characterized using multiple analytical techniques, including FT-IR, Raman spectroscopy, XRD, SEM, TEM, and thermal analysis, confirming the successful integration of graphene and the formation of well-defined nanocomposite structures. The adsorption performance of the [SPVP]TfO-G nanocomposites for Acid Red 1 (AR) dye removal was thoroughly investigated under various experimental conditions. Optimal adsorption was achieved at pH 2 with an adsorbent dosage of 20 mg and contact time of 90 min. Kinetic studies revealed that the adsorption process followed pseudo-second-order kinetics, while thermodynamic analysis indicated the endothermic and spontaneous nature of adsorption. The Langmuir isotherm model best described the adsorption equilibrium, with a maximum adsorption capacity of 21.96 mg g^−1^ for AR dye. The nanocomposites demonstrated excellent performance in removing AR dye from real water samples, including seawater, wastewater, and tap water, with removal efficiencies above 93%. In addition, the nanocomposites exhibited good reusability over four adsorption cycles, highlighting their potential as efficient and sustainable adsorbents for the removal of dye pollutants from aqueous environments.

## Abstract 2

The synthesis and application of a novel nanocomposite material for dye removal from aqueous solutions is described in this work.

## Graphical abstract

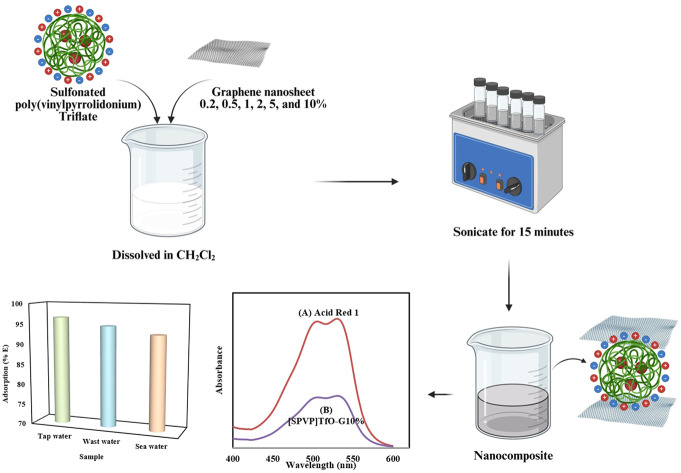

## 1. Introduction

The development of efficient and sustainable methods to remove dyes from wastewater remains an important environmental challenge. Without proper treatment, dyes from various industries can contaminate water sources and pose a risk to aquatic ecosystems and human health. Recent studies have focused on the synthesis and utilization of innovative nanocomposite materials that combine the characteristics of polymers and nanomaterials. These materials are favored owing to their simplicity, cost-effectiveness, and high efficiency, which improve their adsorption capacity and selectivity.^1–8^

In recent years, ionic polymers and their nanocomposites have garnered considerable attention from researchers due to their distinctive properties and potential uses across various domains, including water treatment and dye removal.^9–14^ Sulfonated ionic polymers, in particular, have emerged as promising adsorbents for eliminating organic dyes from aqueous solutions.^15–18^ Numerous studies have investigated the synthesis and characterization of poly(vinylpyrrolidone) (PVP) and its derivatives for various applications.^19–22^

Furthermore, PVP has demonstrated significant potential in water treatment and dye removal applications,^23^ particularly when combined with other materials to form nanocomposites or membranes.^24–26^ For instance, PVP has been successfully used in combination with titanium dioxide nanotubes^27^ and tungsten oxide nanostructures^28^ to develop porous nanocomposite beads for the removal of methylene blue from aqueous solutions. PVP has also been used in combination with other polymers to produce nanofibrous membranes for water filtration. For instance, polyvinylidene fluoride PVDF/PVP-Cu_2_O composite membranes demonstrate high photocatalytic degradation ratios for multiple dyes, including methyl orange, methylene blue, and Congo red.^29^

Incorporating graphene nanosheets into polymer matrices has been shown to imparts unique characteristics to the resulting nanocomposites.^30–32^ The mechanical strength and thermal stability of PVP have been reported to improve in graphene-reinforced PVP nanocomposites^33–35^ making these materials appealing for environmental remediation purposes. When combined with graphene-based materials, sulfonated PVP (SPVP) has been shown to enhance the adsorption capacity and efficiency of composite materials for various dyes, facilitating dye removal from wastewater through adsorption that follows pseudo-second-order kinetics and the Langmuir isotherm model.^36–38^

The selection of counter anions in the synthesis of ionic SPVP significantly influences the adsorption performance of the material in dye removal applications. Triflate anions, known as trifluoromethanesulfonate (TfO), have shown promise for dye removal, especially when integrated into ionic frameworks. Research has explored the integration of TfO anions into ionic liquids, poly(ionic liquids), and polyamide structures with various organic cations to achieve efficient and selective adsorption and elimination of harmful dyes from wastewater.^39–41^

The triflate (TfO^−^) counter-anion significantly influences the surface chemistry of sulfonated poly(vinylpyrrolidonium) (SPVP). Compared with common anions such as Cl^−^, BF_4_^−^, and PF_6_^−^, TfO^−^ possesses a larger ionic radius and a delocalized charge distribution, which modifies the hydration shell and dye-binding sites of SPVP. This alteration promotes enhanced hydrophobic interactions and facilitates stronger π–π stacking and hydrogen bonding between the polymer matrix and aromatic azo and anthraquinone dyes. While electrostatic interactions remain dominant due to the sulfonate groups, the triflate anion synergistically tunes the polymer's surface environment, thereby improving dye adsorption efficiency. Recent studies have underscored the critical role of ionic polymers and nanocomposites in dye removal from contaminated water, emphasizing the importance of counter-anion selection for optimizing adsorbent performance.^42–48^

Although significant progress has been made, additional research is necessary to fine-tune the composition and structure of these composites for specific dye-removal scenarios. This study explored the synthesis and characterization of a new class of nanocomposite materials based on sulfonated poly(vinylpyrrolidonium) triflate [SPVP]TfO, reinforced with varying amounts of graphene nanosheets (G). This study marks the first time that the triflate anion has been examined as a counteranion within the SPVP framework.

The incorporation of both TfO anions and graphene nanostructures into the ionic polymer matrix enhances the adsorption capacity of the material. This study comprehensively characterizes materials using a range of analytical techniques, such as FT-IR, Raman spectroscopy, XRD, SEM, TEM, and thermal analysis. Furthermore, the adsorption performance of the nanocomposites for Acid Red 1 dye (AR dye) removal was assessed by examining factors such as pH, adsorbent dosage, contact time, temperature, and ionic strength. Kinetic and thermodynamic studies were conducted to elucidate the adsorption mechanism and the process efficiency. Finally, the practical applicability of the developed nanocomposites was assessed using real water samples including seawater, wastewater, and tap water.

The results of this research are anticipated to aid to the development of new and effective adsorbents for water treatment and to offer insights into the fundamental properties and behavior of ionic polymer nanocomposites enhanced with graphene nanosheets.

## 2. Results and discussions

### 2.1. Chemistry

**Scheme 1:**
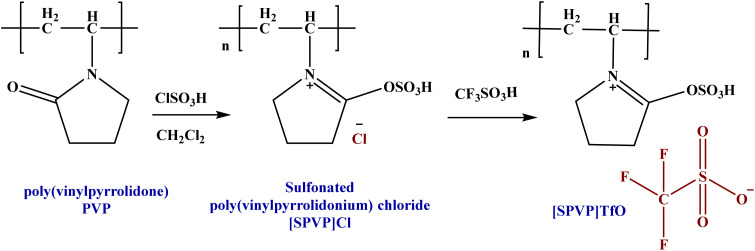
A schematic procedure for synthesizing ionic polymer [SPVP] triflate.

This paper details the synthesis of sulfonated poly(vinylpyrrolidonium) triflate and its nanocomposites reinforced with graphene nanosheets. The process involves two main stages: (1) the formation of the ionic polymer through the sulfonation of polyvinylpyrrolidone and anion exchange with triflate and (2) the incorporation of graphene nanosheets *via* an *in situ* polymerization method.^49^ Initially, [SPVP]Cl was formed using dichloromethane and chlorosulfonic acid, followed by counter-anion substitution with triflate acid to produce [SPVP]TfO (Scheme 1). This novel ionic polymer served as the base material for the subsequent nanocomposite development.

**Scheme 2:**
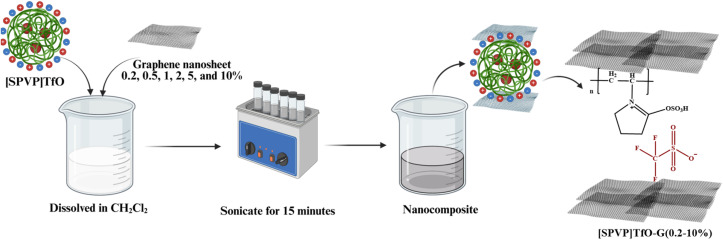
The formulation of [SPVP] triflate loaded with different percentage of graphene nanosheets.

The second stage focused on the integration of graphene nanosheets into the [SPVP]TfO polymer matrix. Using an ultrasonic technique, varying percentages of graphene nanosheets (0.2, 0.5, 1, 2, 5, and 10%) were incorporated into the polymer (Table 1).^50,51^ This resulted in a series of nanocomposites with different graphene contents. An *in situ* polymerization method, similar to that used for the ionic [SPVP]TfO, was employed for nanocomposite synthesis (Scheme 2). The use of ultrasound during this process ensures the uniform dispersion of the nanoparticles throughout the polymer matrix, which is crucial for achieving the desired properties in the final nanocomposite materials.

**Table 1:** Symbols and loading ratios for [SPVP]TfO-G nanocomposites

Composite	Loading percentage of graphene nanosheets
[SPVP]TfO-G0.2%	0.2%
[SPVP]TfO-G0.5%	0.5%
[SPVP]TfO-G1%	1%
[SPVP]TfO-G2%	2%
[SPVP]TfO-G5%	5%
[SPVP]TfO-G10%	10%

### 2.2. IR and Raman analysis

**Fig. 1:**
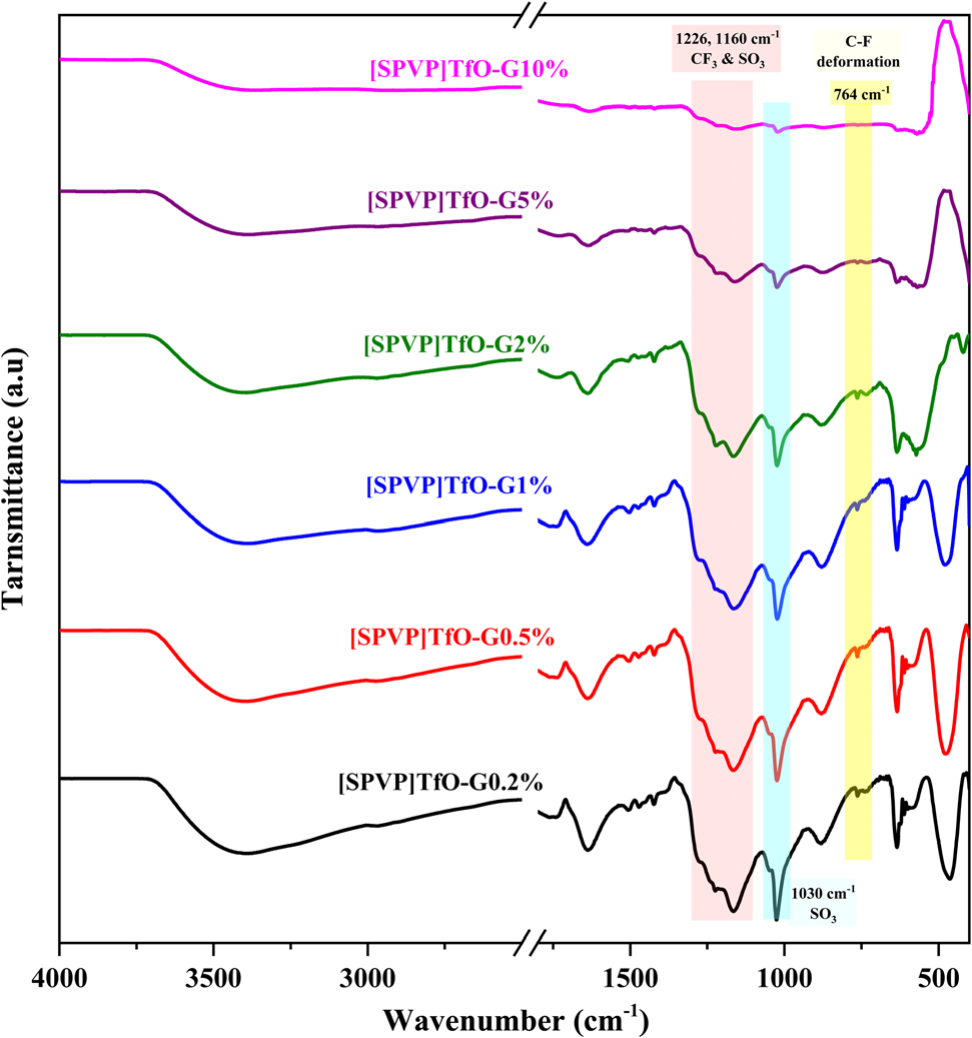
IR spectra of the prepared ionic polymer [SPVP]triflate nanocomposites.

**Fig. 2:**
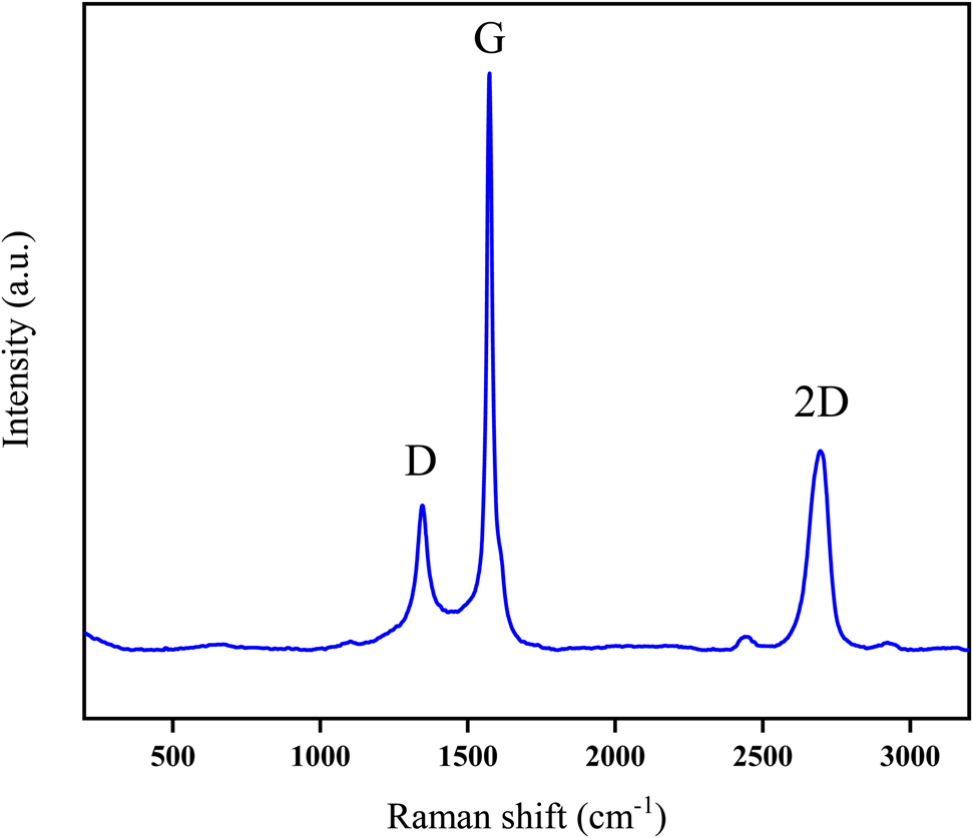
Raman spectra of the designed [SPVP]TfO-G2% nanocomposites.

FT-IR and Raman spectroscopic analyses of the newly fashioned nanocomposites revealed the successful counter anion replacement and the structural and compositional information of the G reinforcement (Fig. 1 and 2). The IR spectra of the triflate ionic polymer [SPVP]TfO-G0.2-10% exhibited characteristic vibrational bands associated with the CF_3_SO_3_^−^ group, providing evidence of their presence in the polymer matrix. The observed stretching vibration at 1160 cm^−1^ for CF_3_ and the bending mode near 764 cm^−1^ confirm the incorporation of the triflate group. Additionally, the asymmetric and symmetric stretching vibrations of the SO_3_ group at 1226 and 1030 cm^−1^, respectively, further supported the successful integration of the triflate ionic polymer complex (Fig. 1).

As the graphene content in the nanocomposites increased from 0.2 to 10%, notable broadening and deformation of the peaks in the ionic polymer complex was observed in the spectra. This peak broadening suggests a significant interaction between the [SPVP] network and graphene nanosheets, indicating the formation of a well-integrated nanocomposite structure.^50,51^ The presence of all the remaining peaks for [SPVP] and [SPVP]Cl, as confirmed in a previous study, further validates the successful synthesis of the nanocomposites.^49^ These spectroscopic findings provide valuable insights into the molecular structure and interactions within the newly created nanocomposites, demonstrating the effectiveness of the synthesis process for combining ionic polymer complexes with graphene nanosheets.

The incorporation of graphene nanosheets into the polymer matrix was further analyzed using Raman spectroscopy, focusing on the [SPVP]TfO-G2% nanocomposite as a representative sample (Fig. 2). The Raman spectra revealed three primary bands of interest: the G-band, D-band, and 2D-band. The G-band observed at 1560 cm^−1^ indicates the presence of in-plane stretching vibrations of sp^2^ bonded carbon atoms. This slight shift from the typical monolayer peak position of 1585 cm^−1^ suggested an increase in the number of graphene layers, as the peak tends to shift to lower wavenumbers with increasing layer count. The intensity of the G-band also increases with the number of layers, providing a useful metric for assessing the layer thickness.^52^

The 2D-band, observed at 2698 cm^−1^, resulted from a double resonance-enhanced two-phonon lateral vibrational process and was also sensitive to graphene folding. An *I*_2D_/*I*_G_ ratio of less than 1 further confirmed the presence of multiple graphene layers in the nanocomposite. The D-band, a narrow and less intense band around 1348 cm^−1^, indicated a lower number of defects and reduced disorder within the carbon lattice of the polymer matrix. These Raman spectroscopy results provide valuable insights into the structural characteristics and quality of graphene nanosheets incorporated into the designed polymer nanocomposite, demonstrating the successful integration of graphene layers with minimal defects.^52^

### 2.3. Morphology study

**Fig. 3:**
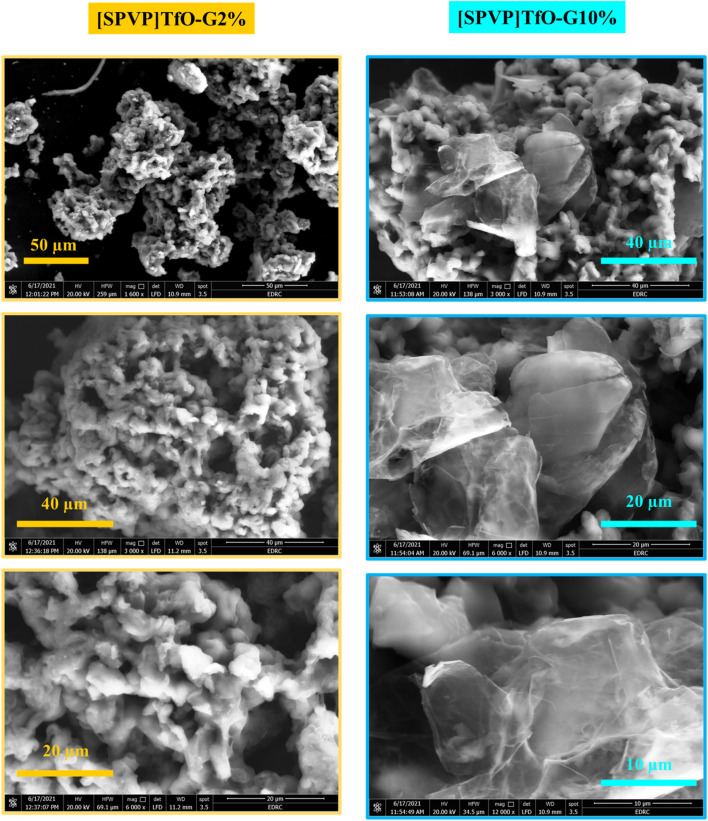
SEM images of [SPVP]TfO-G2% and [SPVP]TfO-G10% nanocomposites.

**Fig. 4:**
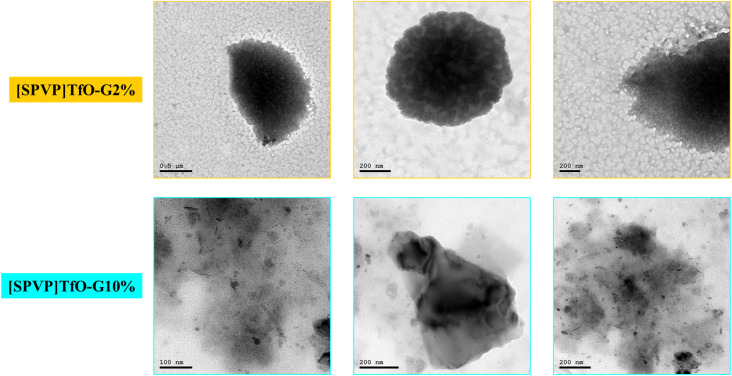
TEM images of [SPVP]TfO-G2% and [SPVP]TfO-G10% nanocomposites.

SEM and TEM analyses of the [SPVP]TfO-G2% and [SPVP]TfO-G10% nanocomposites provided crucial insights into their structural characteristics (Fig. 3 and 4). For [SPVP]TfO-G2%, the SEM images revealed a surface composed of small asymmetrical spheres with varying porosities. These pores became more pronounced at higher magnifications, contributing to the increased surface area and enhanced absorption properties of the polymer.^49^ This structure suggests the potential for improved interactions with the dye molecules, facilitating their removal from the solution. However, the morphology of the [SPVP]TfO-G10% nanocomposite with higher graphene loading showed smooth black graphene sheets layered on the surface of the spheres, indicating the successful deposition and coating of graphene nanosheets. This network/layer structure, characterized by a rough and well-defined topography, is particularly advantageous for nanocomposite functionality.^34,50,51^ The combination of the increased surface area and the presence of graphene sheets likely enhance the capacity of the material to adsorb and remove dye species from solution, potentially improving the overall efficiency of dye removal applications.

The transmission electron microscopy (TEM) analysis of nanocomposites containing 2% and 10% graphene nanosheets within the ionic polymer matrix revealed distinct morphological characteristics (Fig. 4). The images of both [SPVP]TfO-G2% and [SPVP]TfO-G10% nanocomposites exhibit an archetypal sheet-graphene morphology, characterized by a folded, film-like structure dispersed across the ionic polymer surface. This observation confirms the successful incorporation of graphene nanosheets into the network and provides insights into their distribution and interaction with the ionic matrix. A notable difference is observed between the 2% and 10% graphene-loaded nanocomposites. The higher concentration of graphene nanosheets in the 10% nanocomposite resulted in a more noticeable and prevalent sheet-like morphology compared to the 2% nanocomposite.^34,50,51^ This increased visibility of the graphene structures in the 10% nanocomposite suggests a greater degree of graphene dispersion and potentially enhanced interaction with the polymer matrix.

**Fig. 5:**
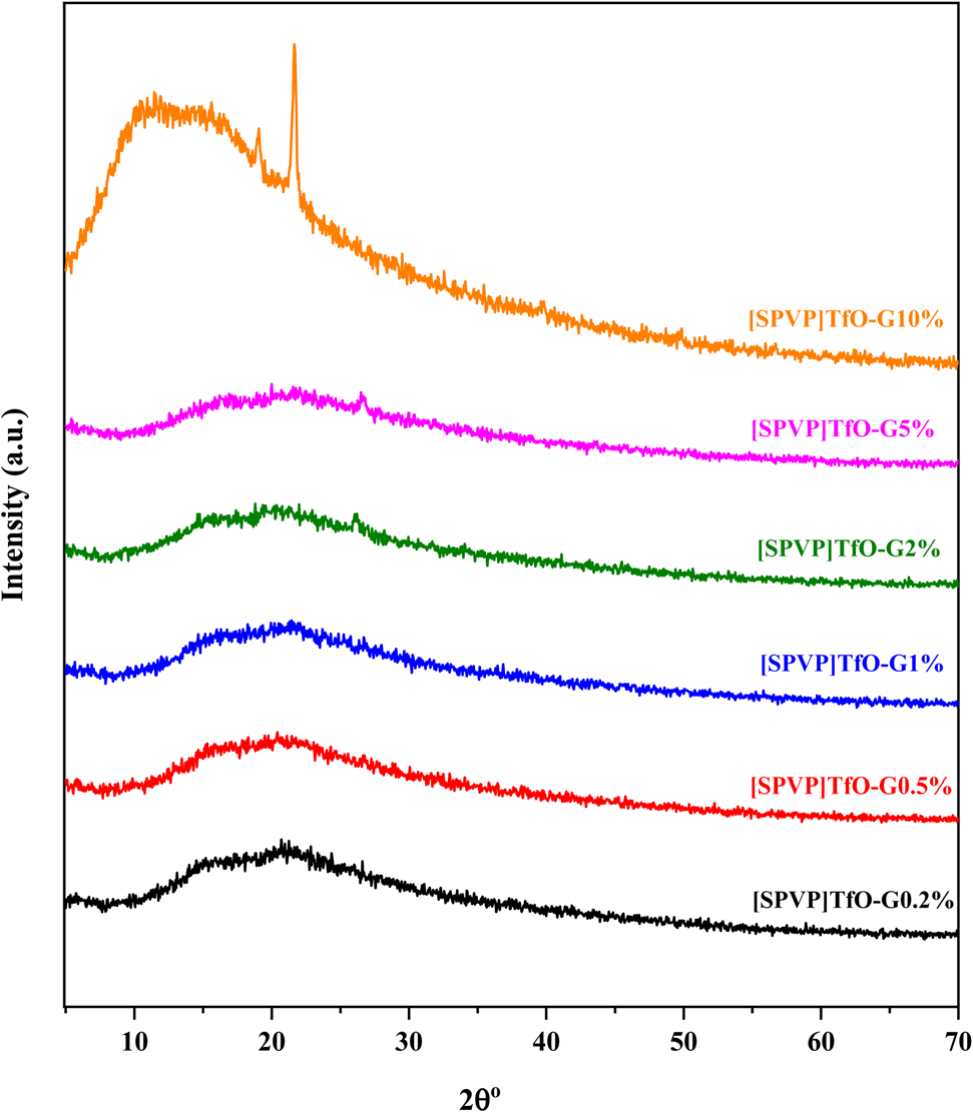
XRD crystallography of [SPVP]TfO-G0.5% to [SPVP]TfO-G10% nanocomposites.

The X-ray diffraction analysis was conducted to provide insights into the integration of nanoparticles within the polymer matrix and the crystalline characteristics of the resulting nanocomposites (Fig. 5). All synthesized ionic polymers exhibited an amorphous microstructure, as evidenced by their crystallographic patterns. A broad diffraction peak was observed in the XRD spectra between 16° and 26°, corresponding to the (002) plane associated with the *d*-spacing between graphitic layers.^49,53^ The intensity of this peak increased progressively as the graphene content increased from 0.2% to 10%, indicating a correlation between the graphene concentration and structural properties of the nanocomposites.^54^ Interestingly, nanocomposites containing 10% nanostructure displayed a crystalline peak at 21°, suggesting that higher graphene loading influences the semicrystalline structure of ionic polymer nanocomposites. This observation implies that the incorporation of graphene at higher concentrations may induce some degree of crystallinity in the otherwise amorphous polymer matrix.

### 2.4. Thermal study

Thermogravimetric analysis (TGA) of [SPVP]triflate nanostructures revealed a complex thermal degradation process that occurred in multiple stages. The initial weight loss observed below 120 °C can be attributed to the evaporation of the residual moisture within the samples. Following this, the primary degradation of the polymer matrix (PDT_main_) occurred rapidly between 185 and 510 °C, resulting in a significant 70% reduction in the polymer weight.^49–51^ The main degradation phase is further divided into two distinct stages. The first stage, occurring between 185 and 250 °C, is associated with the thermal decomposition of the pendant sulfonic groups and triflate counter anions, while the subsequent stage involves the breakdown of the poly(vinylpyrrolidinium) [PVP] matrix.

**Fig. 6:**
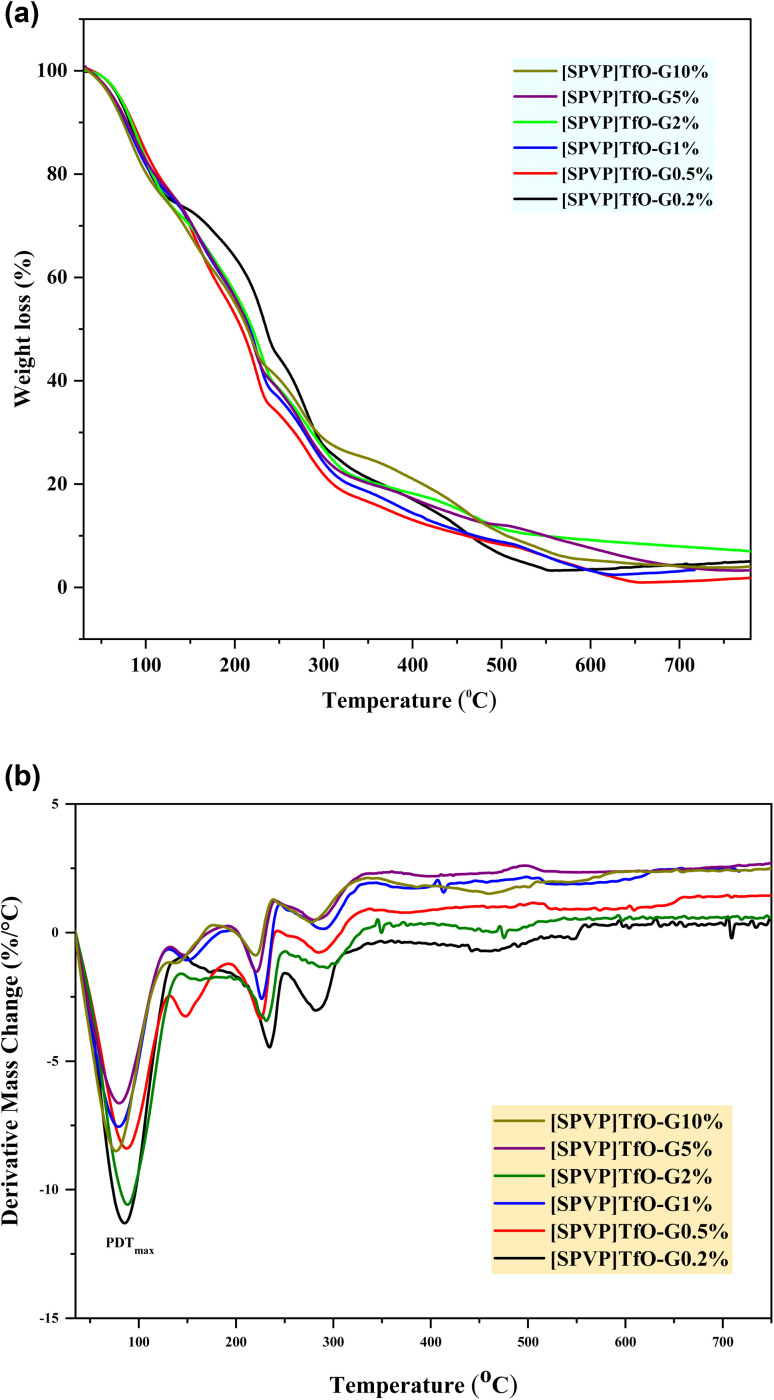
(a) TGA curves of [SPVP]TfO-G0.5% to [SPVP]TfO-G10% nanocomposites. (b) DTG curves of [SPVP]TfO-G0.5% to [SPVP]TfO-G10% nanocomposites.

The incorporation of graphene nanosheets in the nanocomposites, ranging from 0.2% to 10%, led to subtle variations in the thermal behavior of the materials. These differences may be attributed to the influence of graphene on the polymer matrix, which potentially alters the thermal stability and decomposition kinetics of nanocomposites. The observed changes in the thermal characteristics with increasing graphene content suggest that the nanosheets play a role in modifying the overall thermal properties of the [SPVP]triflate nanostructures (Fig. 6a).

The derivative thermogravimetric (DTG) analysis confirms a two-stage degradation process: initial decomposition of pendant sulfonic and triflate groups between 185–250 °C with the maximum degradation temperature of the polymer (PDT_max_) followed by polymer backbone degradation at higher temperatures, supporting the thermal degradation discussion (Fig. 6b).

### 2.5. Removal and adsorption study

The adsorption performance of the nanocomposites was evaluated using Acid Red 1 as the model dye pollutant. Batch adsorption experiments were conducted to examine the effects of various parameters, including pH, adsorbent dosage, contact time, temperature, and ionic strength, on dye removal efficiency.

**Fig. 7:**
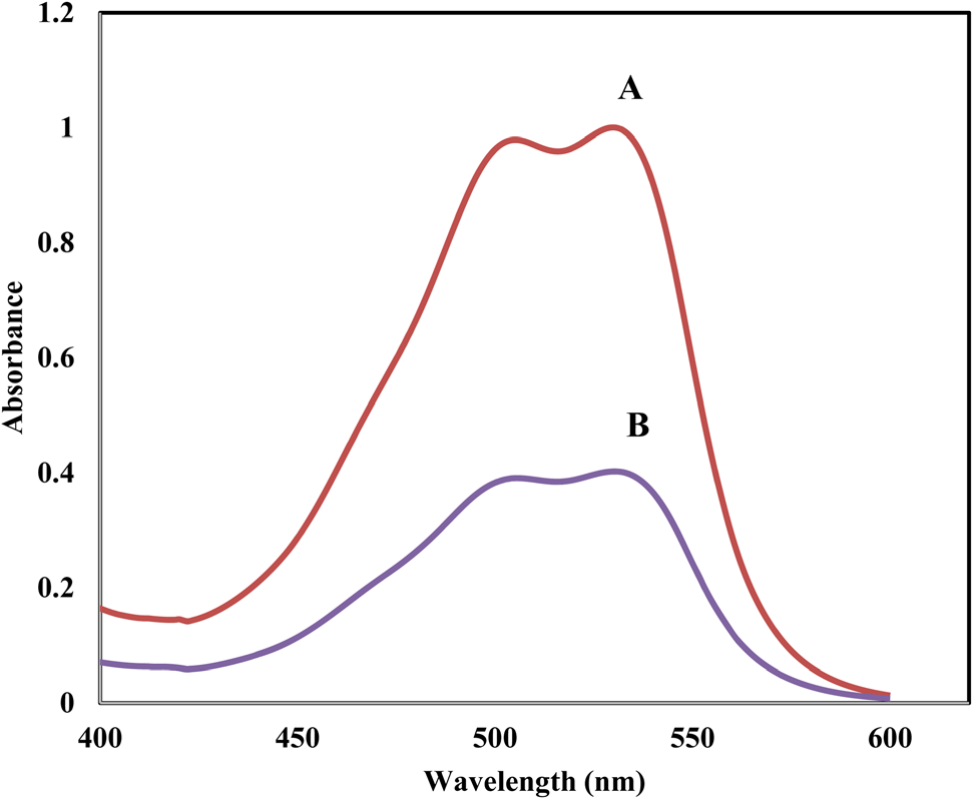
The electronic spectra of 20 ppm concentration of AR dye in aqueous phase (A), and after shaking with 20 mg of formed solid phase (B).

The absorption spectrum of Acid Red 1 in water exhibited a prominent peak at 530 nm, which is characteristic of the molecular structure of this dye and its interaction with light. This peak indicates the dye concentration in the aqueous solution, and serves as a baseline for assessing the effectiveness of the extraction methods (Fig. 7A). When the solution was agitated with [SPVP]TfO-G10%, a notable reduction in the intensity of this peak was observed, signifying a substantial decrease in the concentration of AR dye in water (Fig. 7B).

This significant decrease in the absorption peak demonstrates the high efficacy of the ionic polymer triflate nanocomposites in extracting the AR dye from an aqueous solution. The [SPVP]TfO-G10% nanocomposite likely interacts with dye molecules through various mechanisms, such as electrostatic interactions, π–π stacking, and/or hydrogen bonding. This interaction results in the removal of the dye from the aqueous phase, thereby effectively decolorizing the solution. The ability of these nanocomposites to efficiently extract dyes suggests their potential application in water treatment processes, particularly in industries where dye-contaminated wastewater is a concern.

The combination of TfO^−^ counterions with graphene nanosheets in [SPVP]TfO-G nanocomposites creates synergistic selectivity toward dye classes. TfO^−^ anions enhance electrostatic attraction toward cationic dyes, while graphene nanosheets provide π–π stacking and hydrophobic interactions for neutral and aromatic dyes, leading to improved adsorption efficiency. The G10% nanocomposite was selected for detailed experiments based on SEM and TEM analyses showing optimal graphene dispersion, while adsorption tests confirmed that 10 wt% graphene provided the best balance between adsorption capacity and mechanical stability.

#### 2.5.1. Interactive effect of pH and nanocomposite dosage

**Fig. 8:**
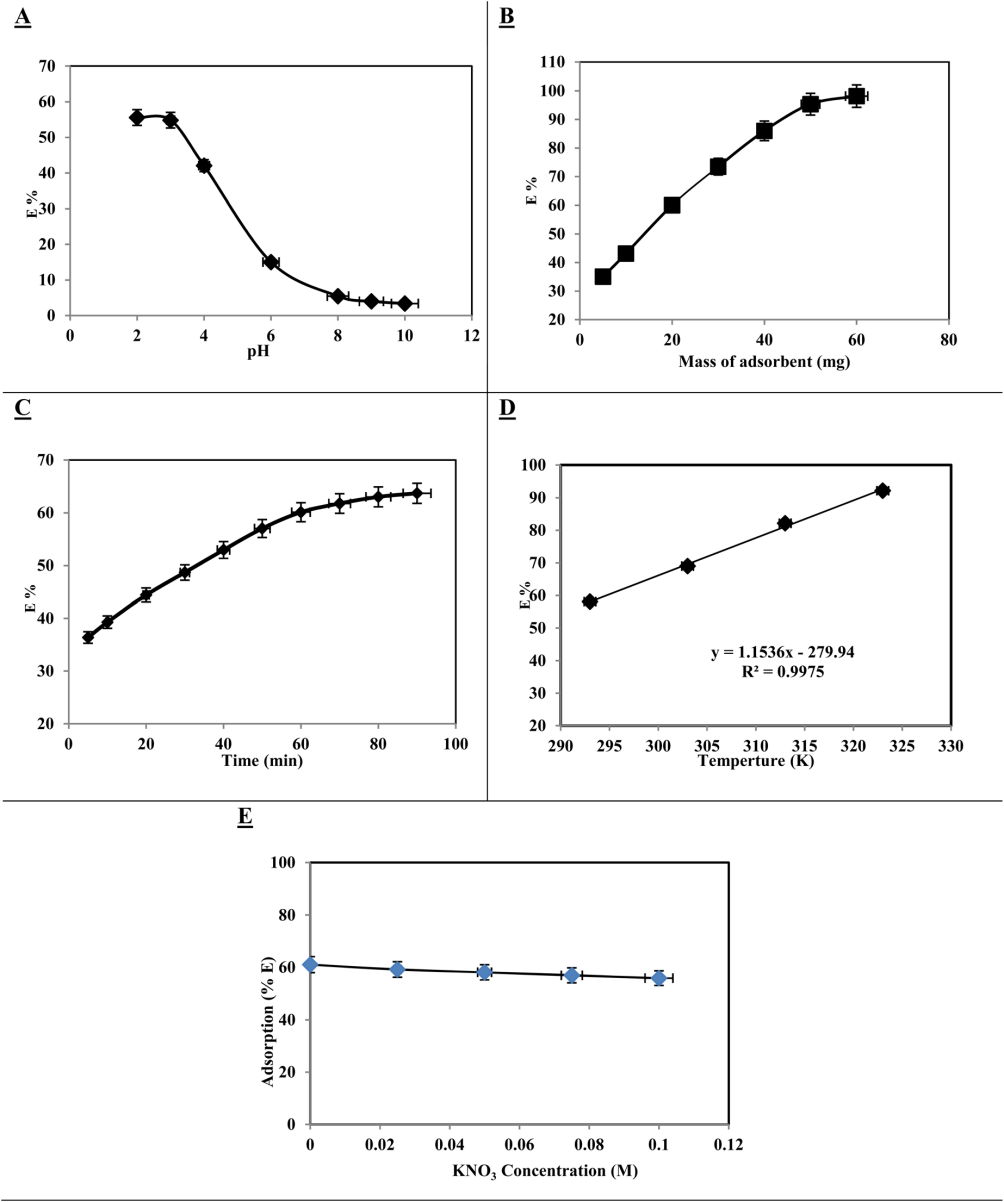
Effect of (A) solution pH, (B) nanocomposite dosage, (C) shaking time, (D) temperature (293, 303, 313, 323 K), and (E) KNO_3_ concentration on the adsorption percentage of Acid Red dye (20 ppm) from aqueous solutions at pH 2 using 20 mg of [SPVP]TfO-G10% nanocomposite with 1 h shaking time at 20 ± 0.1 °C.

The pH of the solution significantly influenced the adsorption of dyes and heavy metals, as demonstrated in the study of AR dye adsorption onto [SPVP]TfO-G10%. This study examined pH levels from 2 to 10 using a NaOH/HCl mixture at room temperature for 120 min (Fig. 8a). The results showed that the highest adsorption percentage occurred at an acidic pH of 2 and the removal efficiency decreased as the pH increased. An optimal pH of 2 was selected for subsequent experiments, highlighting the importance of acidic conditions for effective AR dye removal using the triflate nanocomposite. Several studies have reported optimal adsorption at acidic pH values, likely owing to electrostatic interactions between the positively charged polymer and negatively charged dye molecules.^55,56^

The impact of adsorbent mass on the adsorption process was also investigated (Fig. 8b). As the [SPVP]TfO-G10% dosage increased from 5 mg to 60 mg, the percentage of adsorbed AR dye increased significantly from 35.1% to 98.1%. This trend was attributed to the increased surface area of the ionic polymer nanocomposite, which provided more active adsorption sites. To evaluate other factors affecting adsorption, the researchers chose to use 20 mg of [SPVP]TfO-G10%, which corresponded to a 60% removal rate. These findings underscore the critical role of both the pH and adsorbent dosage in optimizing the adsorption process for dye removal, thereby providing valuable insights for the development of efficient water treatment technologies.

#### 2.5.2. Interactive effect of time, temperature and ionic strength

The adsorption of the AR dye by the [SPVP]TfO-G10% nanocomposite was significantly influenced by the contact time and temperature. This study revealed a two-phase adsorption mechanism: an initial rapid phase followed by a slow diffusion phase. During the first 60 min, the majority of the AR dye was adsorbed and the process reached equilibrium within 90 min. This rapid initial adsorption can be attributed to the transfer of the AR dye from the aqueous solution to the outer surface of the ionic nanocomposite, whereas the subsequent slower phase involved diffusion of the AR dye among the nanocomposite bundles (Fig. 8c).

Temperature also plays a crucial role in the adsorption process. Experiments conducted at 293, 303, 313, and 323 K demonstrated that increasing the solution temperature led to a notable enhancement in the percentage of the AR dye removed by [SPVP]TfO-G10%. This positive correlation between the temperature and adsorption efficiency suggests that the process is endothermic in nature. This endothermic characteristic implies that higher temperatures facilitate the adsorption of AR dye onto the nanocomposite surface, potentially due to the increased molecular mobility and enhanced interaction between the adsorbate and adsorbent at elevated temperatures (Fig. 8d).

The impact of ionic strength on the adsorption of AR dye is a complex phenomenon that involves the interplay of electrostatic interactions between ionic polymer triflate graphene nanosheet surfaces and dye molecules. As the concentration of potassium nitrate (KNO_3_) increased, the ionic strength of the solution changed, leading to different adsorption scenarios. This study revealed that higher KNO_3_ concentrations resulted in a slight decrease in the percentage of the AR dye removed from the solution. This observation can be attributed to the presence of K^+^ cations, which interfere with the adsorption process by competing with the dye molecules for binding sites on the [SPVP]TfO-G10% nanocomposite surface (Fig. 8e).

The relationship between ionic strength and adsorption efficiency is further complicated by the charge characteristics of the adsorbent and adsorbate. The [SPVP]TfO-G10% nanocomposite surface possessed a specific charge distribution that influenced its interaction with AR dye molecules. As the ionic strength increases, the electrostatic interactions between the adsorbent and adsorbate may be either enhanced or diminished depending on the nature of the charges involved. In this case, the presence of K^+^ cations appears to create a screening effect, reducing the attractive forces between the nanocomposite surface and the dye molecules. This screening effect ultimately leads to a decrease in adsorption efficiency, highlighting the importance of considering ionic strength when optimizing adsorption processes for dye removal applications.^56^

#### 2.5.3. Kinetic studies

Kinetic and isotherm studies were performed to elucidate the adsorption mechanism and to determine the maximum adsorption capacity. The kinetic behavior of pollutants, particularly dye molecules, in aqueous solutions using nanocomposite sorbents is a critical area of study for environmental remediation. This process provides valuable insights into the chemical pathways and mechanisms of the adsorption steps, which are essential for understanding and optimizing pollutant removal techniques. The overall transport rate, which is influenced by intraparticle and film diffusion, plays a crucial role in determining the amount of dye retained on the [SPVP]TfO-G10% adsorption surface. A higher transport rate leads to increased dye retention, which ultimately affects the efficiency of the adsorption process.

The Weber–Morris model, as applied to the experimental results, offers a quantitative approach for analyzing the kinetics of dye removal:^57,58^*q*_*t*_ = *R*_d_(*t*)^1/2^ + *C*

**Fig. 9:**
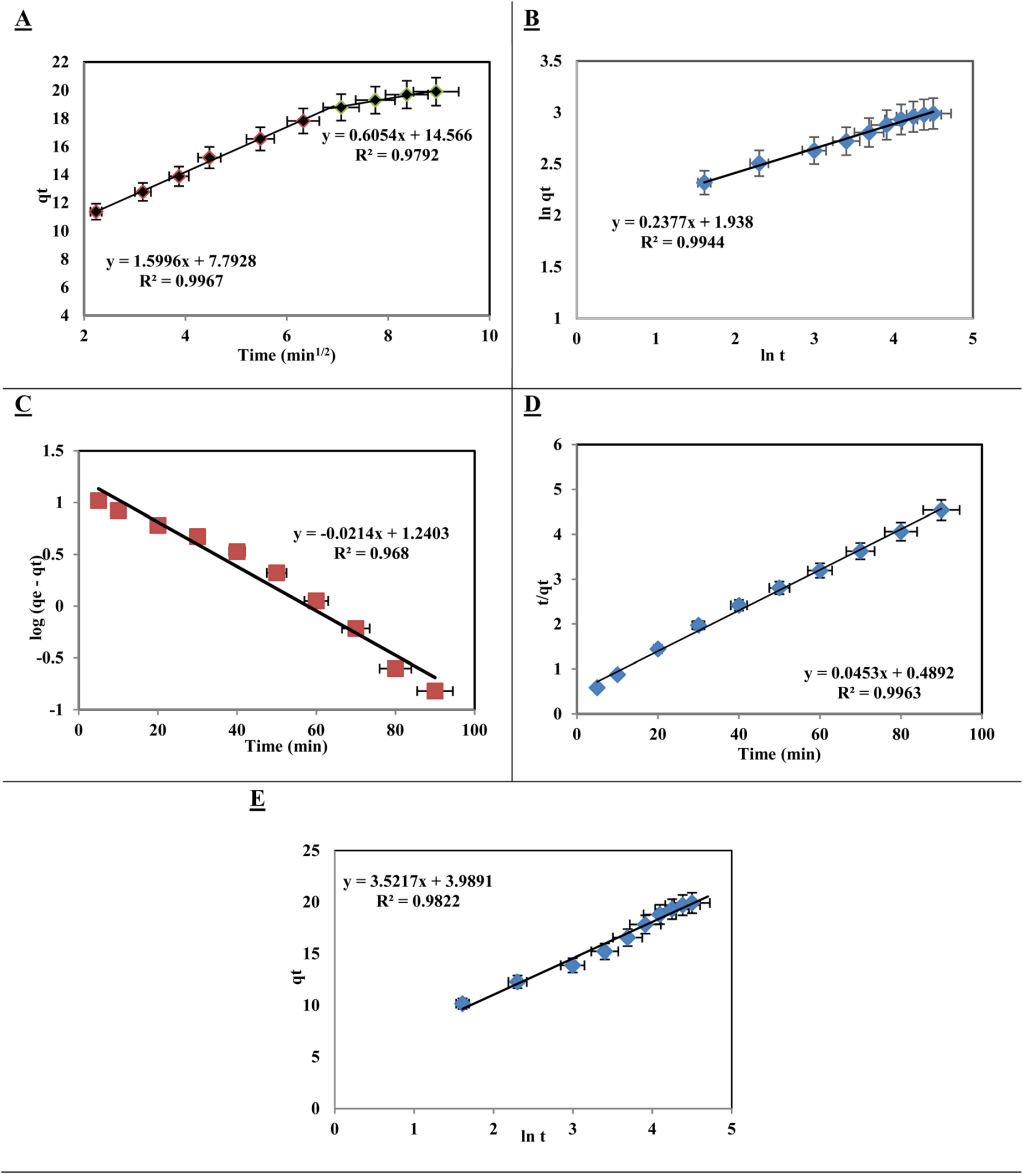
Kinetic model plots for Acid Red dye adsorption onto [SPVP]TfO-G10%: (A) Weber–Morris intraparticle diffusion *versus* square root of time, (B) fractional power function model, (C) Lagergren pseudo-first-order model, (D) pseudo-second-order model, and (E) Elovich model. Experimental conditions: 20 mg adsorbent, 20 ppm dye, 20 °C.

The model parameters, including the rate constant for intraparticle transport (*R*_d_) and the constant percentage of the border-thickness layer (*C*), provided valuable information regarding the adsorption process. The graph of *q*_*t*_*versus* time (Fig. 9a) and the derived *R*_d_ values from the two distinct slopes of the Weber–Morris plots (1.599 and 0.605 mg g^−1^) with high correlation coefficients (*R*^2^ = 0.997 and *R*^2^ = 0.979) indicate a good fit of the experimental data to the model. This analysis suggested the adsorption kinetics and potentially optimized the [SPVP]TfO-G10% nanocomposite sorbent for more effective pollutant removal in water treatment applications.

The kinetic model of the fractional power function is represented by the following equation and provides insights into the adsorption process of the AR dye onto [SPVP]TfO-G10%.^59^ln *q*_*t*_ = ln *a* + *b* ln *t*

The model parameters *a* and *b* are mathematical coefficients, with *b* less than one. When applied to the experimental adsorption data (Fig. 9b), the model showed alignment with the *R*^2^ values for the AR dye. The numerical values of *a* and *b* are presented in Table 2. This alignment suggests that the fractional power function model effectively describes the adsorption behavior of AR dye on the [SPVP]TfO-G10% surface.

The parameters for various kinetic models for removal of AR dye onto [SPVP]TfO-G10% at 20 °C

**Table:** 

Fractional power function kinetic model			
*a*	*b*	*ab*	*R* ^2^
6.945	0.2377	1.651	0.994

**Table:** 

The pseudo-first-order kinetic (Lagergren) model			
*q*_e,exp_ (mg g^−1^)	*q*_e,calc_ (mg g^−1^)	*k*_1_	*R* ^2^
19.91	17.37	0.049	0.968

**Table:** 

The pseudo-second-order kinetic model			
*q*_e,exp_ (mg g^−1^)	*q*_e,calc_(mg g^−1^)	*k*_2_	*R* ^2^
19.91	22.22	4.1 × 10^−3^	0.996

**Table:** 

Elovich kinetic model		
*α*, (g mg^−1^ min^−1^)	*β*, (mg g^−1^ min^−1^)	*R* ^2^
0.882	3.521	0.982

To further investigate adsorption kinetics, the Lagergren equation, commonly used for liquid-phase systems, was applied.^60^ This first-order kinetic model is expressed as
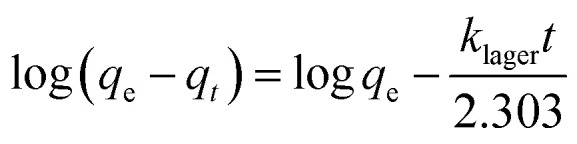
where *q*_e_ is the amount of dye adsorbed at equilibrium, *q*_*t*_ is the amount adsorbed at time *t*, and *K*_lag_ is a first-order rate constant. The plot of log(*q*_e_ − *q*_*t*_) *versus* time (Fig. 9c) shows a linear relationship. However, the calculated values of *K*_lag_, *q*_e_, and *R*^2^ for AR dye removal, listed in Table 2, did not support the first-order kinetic model for the adsorption of AR dye onto ionic polymer triflate nanocomposites. This suggests that the adsorption process may be more complex and potentially governed by other kinetic models or mechanisms.^61^

The pseudo-second-order kinetic model is a widely used approach for describing adsorption processes. This model is based on two key assumptions: the total number of binding sites is determined by the amount of adsorbate at equilibrium and the adsorbate concentration remains constant over time.^62,63^
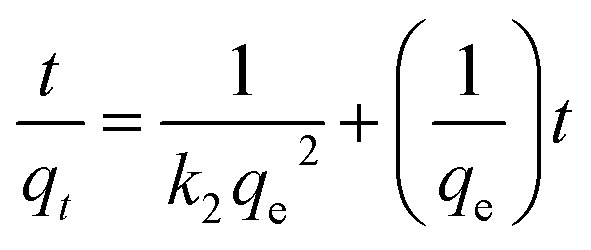


The model is represented by an equation that relates the amount of dye adsorbed per unit weight of adsorbent at equilibrium (*q*_e_) and at any given time (*q*_*t*_), along with the pseudo-second-order coefficient (*k*_2_). When applied to the adsorption of AR dye using triflate nanocomposites, the model demonstrated a good fit, as evidenced by the linear plot of *t/q*_*t*_*versus t*. The intercept and slope of these plots were used to calculate *k*_2_ and *q*_e_ for the dye species, and the results confirmed the suitability of this model for describing the AR dye-removal process (Fig. 9d and Table 2).

In addition to the pseudo-second-order model, the Elovich model was applied to analyze adsorption kinetics.^64^ This model is particularly useful for systems with non-uniform adsorption surfaces and is often associated with chemisorption processes.
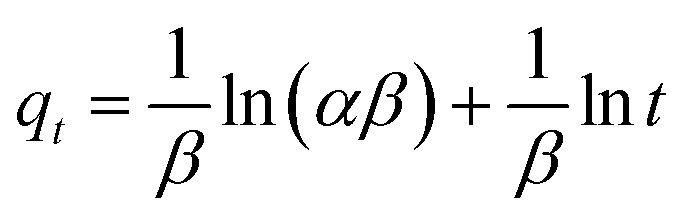


The Elovich equation incorporates the parameters *α* and *β*, which represent the initial adsorption rate and desorption coefficient, respectively. A linear plot of *q*_*t*_*versus* ln *t* was obtained, allowing determination of the *α* and *β* coefficients (Fig. 9e and Table 2). A comparison of the correlation coefficient values and experimental data from various kinetic models, including the Lagergren pseudo-first-order, pseudo-second-order, and Elovich models, revealed that the pseudo-second-order kinetic model provided the best fit for characterizing the adsorption of AR dye onto [SPVP]TfO-G10%. This comprehensive analysis of the adsorption kinetics provides valuable insights into the mechanism and efficiency of the dye removal process using the triflate nanocomposites.

#### 2.5.4. Thermodynamic studies

The thermodynamic characterization of the adsorption of AR dye by an engineered nanocomposite, [SPVP]TfO-G10%, was investigated over a temperature range of 293–338 K. Thermodynamic parameters, including enthalpy (Δ*H*), entropy (Δ*S*), and Gibbs free energy (Δ*G*), were calculated using the following equations: the equilibrium constant (*K*_c_) for dye adsorption was computed using a formula that considered the equilibrium concentrations of AR dye in the water-based solution (*C*_e_) and on the solid surface (*C*_a_) in (mg L^−1^).^65^
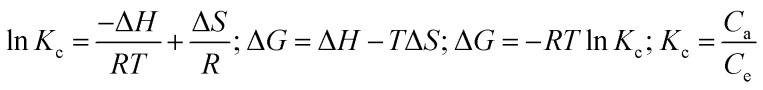


**Fig. 10:**
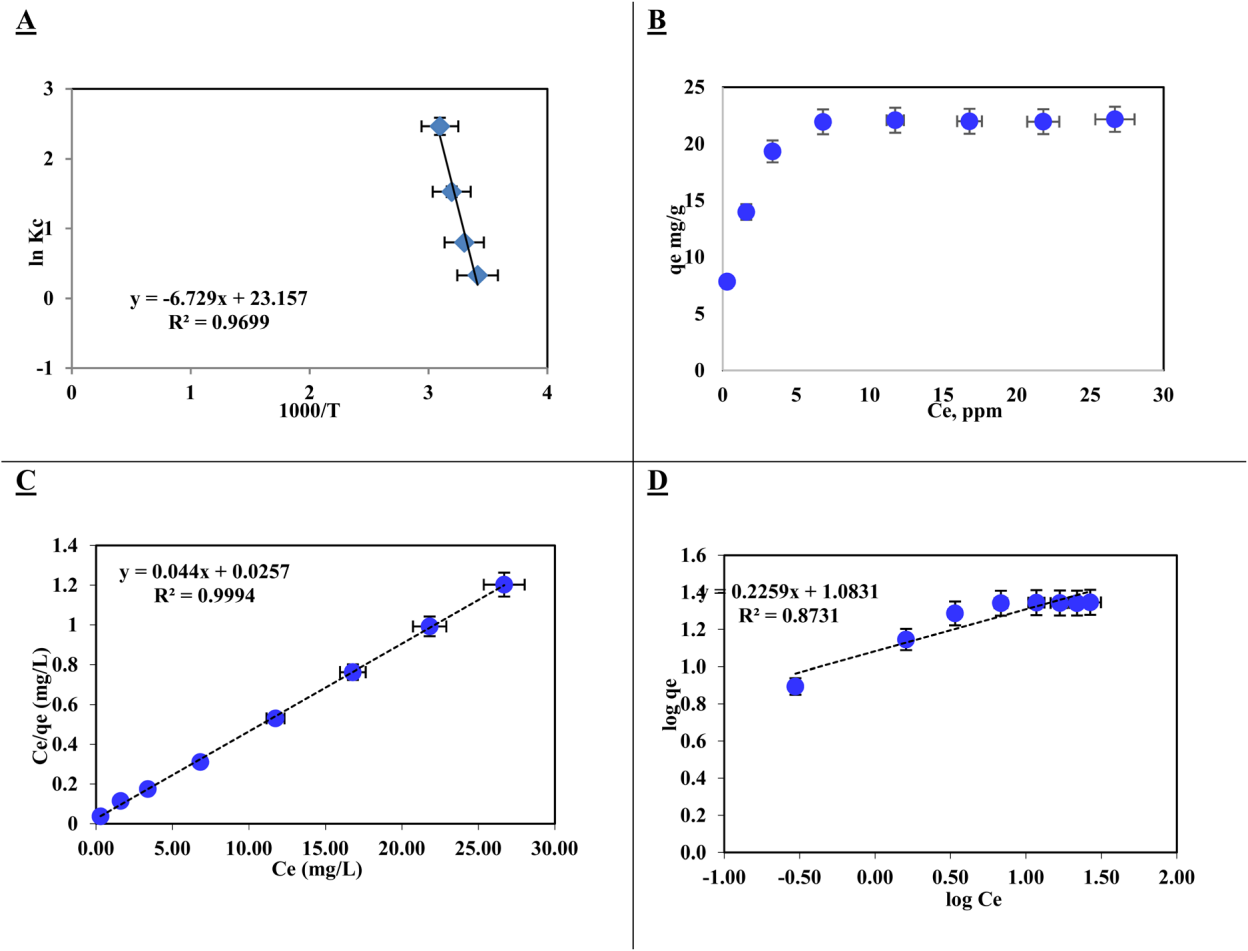
Thermodynamic and isotherm analyses of Acid Red dye adsorption onto [SPVP]TfO-G10% at 20 ± 1 °C: (A) Van 't Hoff plot of ln *K*_c_*versus* 1000/*T*, (B) adsorption capacity (*q*_e_) *versus* equilibrium concentration (*C*_e_), (C) Langmuir isotherm plot (*C*_e_/*q*_e_*versus C*_e_), and (D) Freundlich isotherm plot (log *q*_e_*versus* log *C*_e_).

Analysis of the data revealed a linear relationship between ln *K*_c_ and 1000/*T* for AR dye retention on [SPVP]TfO-G10% within the temperature range – 285–318 K. The increasing equilibrium constant with increasing temperature indicates the endothermic nature of AR dye retention on the sorbents. Calculated thermodynamic values at 293 K showed a positive enthalpy (55.94 ± 0.1 kJ mol^−1^) and entropy (192.5 ± 0.15 J mol^−1^ K^−1^), while Gibbs free energy was negative (−0.46 ± 0.02 kJ mol^−1^). These results provide insights into the thermodynamic behavior of the adsorption process, suggesting a spontaneous and endothermic reaction with increased disorder at the solid–liquid interface, likely due to desolvation effects during dye binding as illustrated in the Van 't Hoff plots (Fig. 10a).

The positive enthalpy (Δ*H*) value of 55.94 ± 0.1 kJ mol^−1^ indicates an endothermic uptake process, suggesting that energy is required to break existing bonds and form new bonds between the dye and the adsorbent. The positive entropy (Δ*S*) value of 192.5 ± 0.15 J mol^−1^ K^−1^ implies increased randomness at the solid–liquid interface, likely due to the release of water molecules from the hydration sphere during adsorption. The negative Gibbs free energy (Δ*G*) value of −0.46 ± 0.02 kJ mol^−1^ at 293 K confirms the spontaneous nature of the adsorption process.

In addition, the adsorption isotherms provided valuable insights into the interaction mechanism between the AR dye and [SPVP]TfO-G10% sorbent surface. Equilibrium studies are crucial for determining the maximum adsorption capacity and identifying the key surface properties of sorbents. The retention profiles were analyzed across a range of equilibrium concentrations (5–40 mg L^−1^) under optimal conditions. The relationship between the amount of AR dye retained on [SPVP]TfO-G10% and the equilibrium concentration in the solution exhibits a linear trend at moderate analyte concentrations. The adsorption capacity of AR dye onto [SPVP]TfO-G10% was found to be 21.96 mg g^−1^ (Fig. 10b).

The Langmuir isotherm model was used to analyze the retention of the AR dye on the [SPVP]TfO-G10% sorbent.^66^
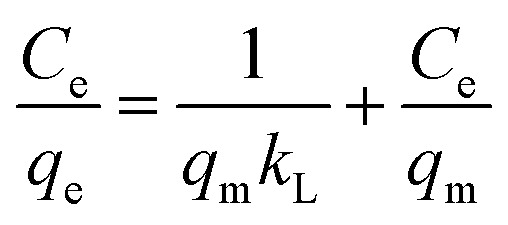
where *C*_e_ represents the equilibrium concentration (mg L^−1^) of the AR dye in the solution being tested and *q*_e_ denotes the quantity of solute adsorbed per unit mass of the adsorbent at equilibrium (mg g^−1^). The constants *q*_m_ and *k*_L_ are Langmuir parameters, where *q*_m_ is the maximum adsorption capacity of the solute per unit mass of adsorbent necessary for monolayer surface coverage, and *k*_L_ is the equilibrium constant associated with the binding energy of solute sorption, which remains constant regardless of the temperature.

The plot of *C*_e_/*q*_e_ against *C*_e_ exhibited a linear relationship with an *R*^2^ value of 0.999, indicating uniform adsorption of the AR dye on the [SPVP]TfO-G10% surface and good alignment with the Langmuir model. The Langmuir parameters, *q*_m_ and *k*_L_, were derived from the slope and intercept of the linear graph, respectively (Fig. 10c and Table 3). The dimensionless separation factor *R*_L_, a crucial feature of this model, was calculated as 0.079 (0 < *R*_L_ < 1), suggesting favorable adsorption conditions for the AR dye onto the [SPVP]TfO-G10% sorbent.^67^
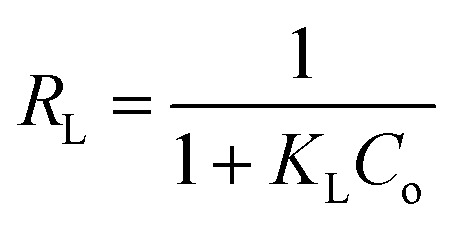


**Table 3:** Isotherm parameters of Langmuir and Freundlich models for AR dye retention onto [SPVP]TfO-G10% at 20 ± 1 °C

Sorption isotherms models		Values
Langmuir	*q*_m_, mg g^−1^	22.73
	*K*_L_, L g^−1^	0.584
	*R*_L_	0.079
	*R* ^2^	0.999
Freundlich	*K*_F_, mg g^−1^	1.083
	1/*n*	0.226
	*R* ^2^	0.873

The Freundlich model is a widely used adsorption isotherm that describes the relationship between the amount of adsorbate (in this case, AR dye) retained on the adsorbent surface and its concentration in the solution at equilibrium.^68^
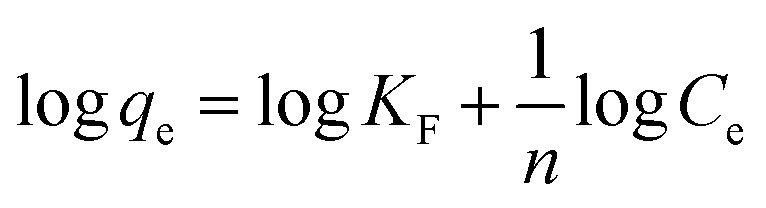


This model assumes a heterogeneous surface with multiple adsorption sites of varying energy. In this study, the Freundlich parameters *K*_F_ and 1/*n* were determined from the intercept and slope of the linear plot (Fig. 10d and Table 3). The *K*_F_ value represents the adsorption capacity and 1/*n* is the adsorption intensity or surface heterogeneity.

The obtained 1/*n* value of 0.226, which is less than 1, suggests that the [SPVP]TfO-G10% sorbent surface is heterogeneous, which is favorable for the adsorption of AR dye. This heterogeneity implies that the adsorption sites have different affinities for the dye molecules, potentially leading to a more efficient removal process. However, the correlation coefficient (*R*^2^) for the Freundlich model was 0.873, which, while indicating a reasonable fit, was lower than that of the Langmuir model. This suggests that the Langmuir model, which assumes monolayer adsorption on a homogeneous surface, provides a better description of the AR dye adsorption process on [SPVP]TfO-G10%. The superior fit of the Langmuir model may indicate that despite the surface heterogeneity, the adsorption process predominantly occurs through the formation of a monolayer on the adsorbent surface.

#### 2.5.5. Environmental real sample test

**Fig. 11:**
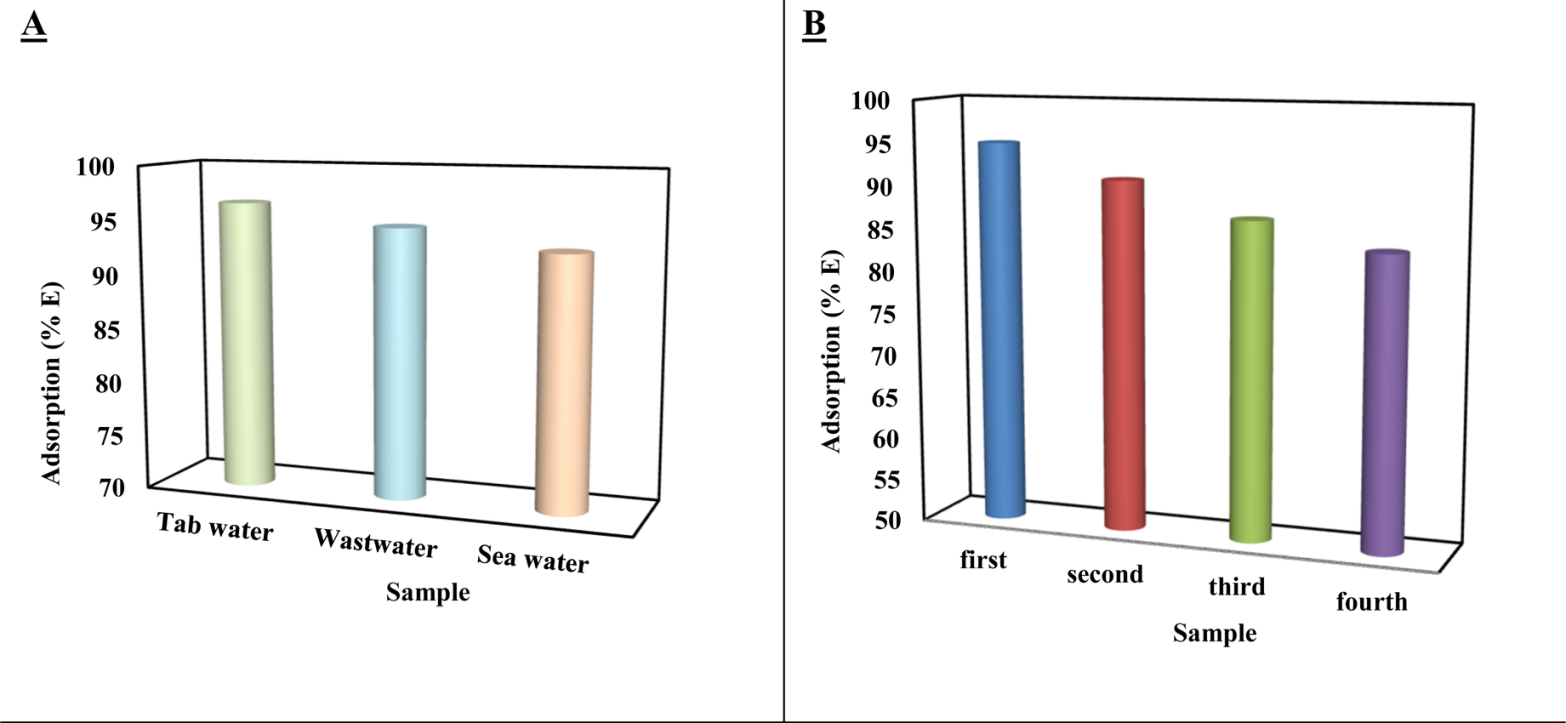
(A) Removal efficiency of Acid Red dye by [SPVP]TfO-G10% from seawater, wastewater, and tap water samples spiked with 20 mg L^−1^ dye at pH 2; (B) reusability performance of [SPVP]TfO-G10% over four adsorption–desorption cycles.

The efficiency of [SPVP]TfO-G10% in adsorbing and eliminating dye species from aqueous solutions was tested using three real water samples: seawater, wastewater, and tap water. Initially, the amount of AR dye in these samples was below the UV-vis detection limit. After spiking the samples with 20 mg L^−1^ of AR dye and adjusting the pH to 2, 60 mg of [SPVP]TfO-G10% was added and the mixture was agitated for 120 min at 303 K. The results showed high dye removal percentages of 93.05%, 94.76%, and 96.57% for seawater, wastewater, and tap water samples, respectively (Fig. 11a).

Real-water adsorption tests were conducted at pH 2 to maximize dye removal efficiency. However, acidification to pH 2 may not be feasible for large-scale field applications. Additional experiments at native pH values of seawater, wastewater, and tap water (∼7) revealed a moderate decrease in adsorption efficiency by approximately 10–15% (Table 4). Despite this reduction, the nanocomposite maintains significant dye uptake, demonstrating practical applicability without stringent pH adjustment.

**Table 4:** Effect of native pH on Acid Red dye removal efficiency by [SPVP]TfO-G10% in real water samples

Water sample	Native pH	Removal efficiency (%) at pH 2	Removal efficiency (%) at native pH
Seawater	∼8	93.05	∼80
Wastewater	∼7	94.76	∼82
Tap water	∼7	96.57	∼85

Subsequently, to assess the reusability of [SPVP]TfO-G10%, the adsorbent was retrieved, washed with acetone to remove the dye, dried, and reused over multiple adsorption cycles. The results demonstrated that [SPVP]TfO-G10% maintained a nearly consistent adsorption efficiency (%*E*) over four cycles (Fig. 11b). This finding indicates that the ionic polymer designed with triflate counter anions and graphene nanosheets can be effectively recycled and reused multiple times without significant loss in its adsorption capacity. The ability to maintain high adsorption efficiency over multiple cycles highlights the potential of [SPVP]TfO-G10% as a sustainable and cost-effective solution for dye removal from various water sources (Table 5).

**Table 5:** Benchmarking comparative table

Adsorbent material	Adsorption capacity (mg g^−1^)	Contact time (min)	Optimal pH	Regeneration cycles	Remarks	Ref.
[SPVP]TfO-G10% nanocomposite	21.96	90	2	4	High removal efficiency; good reusability	This study
Activated carbon	15–30	90–120	4–6	3–5	Widely used; moderate regeneration cost	70 and 71
Commercial resin (*e.g.*, Amberlite)	20–25	90–120	3–5	5	Effective for cationic dyes	72 and 73
Polymer–graphene composite (recent study)	18–23	60–120	2–4	3–4	Enhanced π–π interactions; selective adsorption	74–77

## 3. Experimental

### 3.1. Materials

Organic solvents were sourced from commercial suppliers and utilized in their original form unless otherwise specified. Other chemicals were obtained from Merck, Aldrich, or Acros, and were used without additional purification. In this study, powdered polyvinylpyrrolidone (PVP) was employed to modify the nanocomposites as a matrix, while graphene nanosheets (G) served as a reinforcement. Dichloromethane (CH_2_Cl_2_), ethyl acetate (C_4_H_8_O_2_), and ethanol (C_2_H_5_OH) were used in the preparation of PVP, and chlorosulfonic acid (HSO_3_Cl) along with triflate acid (CF_3_SO_3_H) were utilized for the chemical modification of PVP.

A stock solution of Acid Red 1 at a concentration of 500 µg mL^−1^ was prepared using chemicals from Aldrich Chemical Co. Ltd (Milwaukee, WC, USA). This stock solution was then diluted with deionized water to create standard solutions ranging from 10 to 100 µg mL^−1^. For the sorption process of Acid Red using modified composites, a series of Britton–Robinson (BR) buffers with pH values from 2 to 11 and 0.5 mol per L HCl were utilized as the extraction medium.

### 3.2. Apparatus

Various instruments were employed to characterize and identify the chemically modified PVP and nanocomposite materials. The FT-IR spectra of all the nanocomposites were recorded using a PerkinElmer Spectrum100 FT-IR spectrometer, covering a range of 4000–450 cm^−1^. X-ray diffraction patterns for all resulting nanocomposites were obtained using a D8 ADVANCE X-ray diffractometer, with 2*θ* ranging from 5° to 80°. The thermal properties were assessed by thermogravimetric analysis (TGA) and derivative thermal gravimetric analysis (DTG) using a Shimadzu TG-50H thermal analyzer. Scanning electron microscopy (SEM) was conducted using a JSM-7610FPlus Schottky (JEOL-Japan) to examine the surface morphology supported by EDX. Transmission electron microscopy (TEM) was performed using a JEOL JEM-F200 multipurpose instrument operating at 200 kV. Raman spectroscopy, including 3D and 2D confocal Raman images and Raman shift curve measurements, was carried out using a Raman spectrometer (Lab. RAM-HR Evolution HORIBA France SAS Co.). The sorption progress of Acid Red was measured using a PerkinElmer UV-visible spectrophotometer (model Lambda 25, USA) equipped with a quartz cell with a 10 mm path width, covering a wavelength range of 190–1100 nm. An Orion pH meter (Model EA 940) was used for pH measurements and testing solutions, and a digital micropipette (Volac) was employed to prepare the standard Acid Red solutions. A digital sensitive balance (Citizen Scales Inc., USA) with a precision of up to four decimal places was used. Deionized water sourced from a Milli-Q Plus system (Millipore, Bedford, MA, USA) was used to prepare the solutions.

### 3.3. Standard protocols for preparing modified sulfonated poly(vinylpyrrolidonium) (SPVP)

#### 3.3.1. Preparation of [SPVP]chloride

The ionic polymer [SPVP]Cl was synthesized by dissolving 2 g PVP in 10 mL dichloromethane to create a suspension. Chlorosulfuric acid (1.2 mL) was added to the suspension and the mixture was stirred for 3 h at room temperature. Ethyl acetate was used to filter and wash the mixture, which was subsequently heated to 80 °C. This process was continued until yellow [SPVP]Cl powder was obtained (Scheme 1, later).

#### 3.3.2. Preparation of [SPVP]triflate

A mixture of [SPVP]Cl (3 g) and dichloromethane (20 mL) was prepared, followed by the gradual addition of 0.8 mL of triflate acid with continuous stirring for 15 min in an ice bath. The mixture was subsequently allowed to reach room temperature and heated under reflux for 8 h. Thereafter, the solution was left at room temperature overnight to facilitate solvent evaporation, resulting in the formation of a white powder of the triflate ionic polymer [SPVP]TfO.

#### 3.3.3. Graphene sheets fabricated protocol of [SPVP]TfO nanocomposites

The synthesis of nanocomposites through *in situ* polymerization is a sophisticated approach that allows for the uniform dispersion of graphene sheets within the polymer matrix. In this process, various concentrations of graphene sheets (G) were incorporated, ranging from 0.2%, 0.5, 1, 2, 5, and 10%, based on the weight percentage of [SPVP]TfO. This wide range of concentrations enables researchers to study the effect of the graphene content on the properties of the resulting nanocomposites. Ultrasonication for 15 min at room temperature is a critical step in the synthesis process because it helps achieve a homogeneous dispersion of graphene sheets within the polymer precursor solution (Scheme 2).

#### 3.3.4. Batch extraction step

A precise weight (0.02 ± 0.003 g) of [SPVP]TfO-G10% nanocomposite was balanced with a 20 mL aqueous solution containing Acid Red (AR) at a concentration of 20 mg L^−1^, in the presence of HCl at 0.1 mol L^−1^. The solution was agitated for one hour on a mechanical shaker. Subsequently, the aqueous phase was separated and the remaining AR in this phase was measured photometrically against a reagent blank.^69^ The amount of AR absorbed by [SPVP]TfO-G10% was determined by calculating the difference in the absorbance of AR in the aqueous phase before (*A*_b_) and after (*A*_f_) extraction. The sorption percentage (%*E*), equilibrium amount of AR retained per unit mass of solid sorbent (*q*_e_) in mol g^−1^, and distribution coefficient (*K*_d_) of the sorbed analyte on [SPVP]TfO-G10% were calculated, as previously described. The %*E* and *K*_d_ values represent the averages of three separate measurements with a precision of ±3% in most instances. Using these methods, the effects of shaking time and temperature on AR retention by [SPVP]TfO-G10% sorbent were examined.

#### 3.3.5. Environmental applications and sample collection

Samples of Red Sea water, wastewater, and tap water were used to assess the effectiveness of the [SPVP]TfO-G10% sorbents in extracting and recovering Acid Red dye. Red Sea water was sourced from the Red Sea near Jeddah City, KSA, and the wastewater sample was obtained from a wastewater treatment facility at King Abdulaziz University, Jeddah City, KSA. Tap water was obtained from Chemistry Department Laboratories at King Abdulaziz University, Jeddah City, KSA. The samples were filtered through a 0.45 µm membrane and stored in Teflon bottles at 5 °C in a dark environment. A 100 mL sample was adjusted to a pH of 2 using HCl (0.5 mol L^−1^) and then passed through the nanocomposite. The quantity of dye recovered was determined using spectrophotometry.

## 4. Conclusion

In this study, a novel nanocomposite material based on sulfonated poly(vinylpyrrolidonium) triflate [SPVP]TfO reinforced with graphene nanosheets was developed for the efficient removal of Acid Red 1 dye from aqueous solutions. FT-IR, Raman spectroscopy, XRD, SEM, TEM, and thermal analysis confirmed the successful integration of graphene and the formation of well-defined nanocomposite structures. The optimal adsorption conditions were determined to be a pH of 2, adsorbent dosage of 20 mg, and contact time of 90 min. The adsorption process followed pseudo-second-order kinetics and was best described by the Langmuir isotherm model, with a maximum adsorption capacity of 21.96 mg g^−1^ for AR dye. Thermodynamic analysis indicated that the adsorption process was endothermic and spontaneous in nature. The nanocomposites demonstrated excellent performance in removing AR dye from real water samples (seawater, wastewater, and tap water) with removal efficiencies above 93%. Good reusability was observed over the four adsorption cycles, highlighting its potential for sustainable applications. The [SPVP]TfO-graphene nanocomposites developed in this study show great promise as efficient and reusable adsorbents for the removal of anionic dyes from aqueous environments. The combination of the ionic polymer matrix and graphene nanosheets resulted in enhanced adsorption properties compared with those of conventional materials. Further optimization and scale-up studies are recommended to fully realize the potential of these nanocomposites for large-scale water-treatment applications.

## Conflicts of interest

There are no conflicts to declare.

## Data Availability

The authors confirm that all data are included in the manuscript.
